# Paraoxonase-1 Concentrations in Obstructive Sleep Apnoea: A Systematic Review and Meta-Analysis

**DOI:** 10.3390/antiox11040766

**Published:** 2022-04-12

**Authors:** Maria Carmina Pau, Angelo Zinellu, Elisabetta Zinellu, Gianfranco Pintus, Ciriaco Carru, Alessandro G. Fois, Arduino A. Mangoni, Pietro Pirina

**Affiliations:** 1 Department of Medical, Surgical and Experimental Sciences, University of Sassari, 07100 Sassari, Italy; mcpau@uniss.it (M.C.P.); agfois@uniss.it (A.G.F.); 2 Department of Biomedical Sciences, University of Sassari, 07100 Sassari, Italy; azinellu@uniss.it (A.Z.); gpintus@uniss.it (G.P.); carru@uniss.it (C.C.); 3 Clinical and Interventional Pneumology, University Hospital Sassari (AOU), 07100 Sassari, Italy; elisabetta.zinellu@aousassari.it; 4 Department of Medical Laboratory Sciences, Sharjah Institute for Medical Research, College of Health Sciences, University of Sharjah, Sharjah 27272, United Arab Emirates; 5 Quality Control Unit, University Hospital of Sassari (AOU), Viale San Pietro, 07100 Sassari, Italy; 6 Flinders Medical Centre, Department of Clinical Pharmacology, College of Medicine and Public Health, Flinders University, Bedford Park, SA 5042, Australia; arduino.mangoni@flinders.edu.au

**Keywords:** obstructive sleep apnoea, sleep disorder, paraoxonase-1(PON-1), oxidant–antioxidant imbalance

## Abstract

Obstructive sleep apnoea (OSA) is characterized by overproduction of reactive oxygen species and oxidative stress. The antioxidant enzyme paraoxonase-1 (PON-1) may be useful for monitoring the antioxidant defence systems and the effect of treatments in OSA patients. We investigated, by means of systematic review and meta-analysis, the serum concentrations of PON-1 in OSA patients and non-OSA controls. A literature search was conducted in PubMed, Web of Science, Scopus and Google Scholar databases, from the outset to November 2021, utilizing the terms: “paraoxonase” or “PON” or “paraoxonase-1” or “PON-1” and “obstructive sleep apnoea syndrome” or “OSAS” or “OSA”. Eleven studies in 429 OSA patients and 258 non-OSA controls were involved in the meta-analysis. The pooled serum PON-1 concentrations were significantly lower in OSA (standardized mean difference (SMD) = −0.70, 95% CI −1.13 to −0.28; *p* = 0.001). Despite the extreme between-study heterogeneity, the SMD values were not substantially affected by the sequential omission of individual studies. There was no publication bias. Our systematic review and meta-analysis supports the presence of an impaired antioxidant defence system in OSA, possibly the consequence of intermittent hypoxia. Further studies are required to determine the clinical use of PON-1 measurements for risk stratification and monitoring in OSA patients.

## 1. Introduction

Obstructive sleep apnoea (OSA) is a respiratory disorder that occurs with recurrent episodes of upper airway blockage during sleep [1]. These events are associated with oxyhemoglobin desaturations and are considered equivalent to hypoxia-reperfusion injury, a condition that enhances reactive oxygen species (ROS) production and inflammatory response [2]. OSA is known to be associated with obesity, hyperglycaemia and dyslipidaemia, and is an independent risk factor for atherosclerotic cardiovascular disease [3].

It has been proposed the over-production of ROS and the impairment in antioxidant defence systems might account for the development and the progression of atherosclerosis in OSA [4]. There are various known antioxidant molecules which play a defensive role against excess oxidative stress in different diseases [5,6]. Paraoxonases (PON) are a family of enzymes that protect against oxidative damage and lipid peroxidation, modulating the susceptibility of high-density cholesterol lipoprotein (HDL) to atherogenic modifications [7,8]. The (PON) family includes PON-1, PON-2 and PON-3. PON-1 and PON-3 bind to circulating HDL, while PON-2 is located intracellularly [9]. PON-1 and its isoforms, the most studied, are involved in the prevention of cardiovascular and neurodegenerative diseases [10,11,12]. Alterations of PON-1 activity have been reported in various inflammatory and oxidative stress-related diseases, e.g., type 2 diabetes, chronic obstructive pulmonary disease and sleep disorders [13,14,15]. However, conflicting results have been reported regarding the association between PON-1 and OSA [16,17]. We sought to address this issue by critically appraising, by means of systematic review and meta-analysis, studies investigating serum PON-1 concentrations in OSA patients and non-OSA controls.

## 2. Materials and Methods

### 2.1. Search Strategy, Eligibility Criteria and Study Selection

A systematic literature search of publications in the databases PubMed, Web of Science, Scopus and Google Scholar, from the outset to November 2021, was conducted using the terms “paraoxonase” or “PON” or “paraoxonase-1” or “PON-1” and “obstructive sleep apnoea syndrome” or “OSAS” or “OSA”, and their combination. The abstracts were independently examined by two investigators, who revised only the relevant complete articles. Eligibility criteria were: (i) Evaluation of PON-1 activity in plasma or serum; (ii) comparison between patients with OSA and non-OSA subjects (case–control design); (iii) sample size ≥10 OSA patients; (iv) English language; and (v) the presence of full text. To identify additional studies, the references of the recovered articles were also examined. The presence of any disagreement between reviewers was resolved involving a third investigator.

The Joanna Briggs Institute (JBI) Critical Appraisal Checklist for analytical studies was utilized to assess the risk of bias. A score of ≥5, 4 and <4 indicated low, moderate and high risk, respectively [18]. The Grades of Recommendation, Assessment, Development and Evaluation (GRADE) Working Group system was used to evaluate the certainty of evidence. GRADE considers the study design (randomized vs. observational), the existence of unexplained heterogeneity, the risk of bias (JBI checklist), the indirectness of evidence, the presence of imprecise results (sample size, 95% confidence interval width and threshold crossing), the effect size (standardized mean difference (SMD) < 0.5, small; SMD 0.5–0.8, moderate; and SMD > 0.8, large) [19] and the probability of publication bias [20,21]. The study fully conforms with the Preferred Reporting Items for Systematic reviews and Meta-Analyses (PRISMA) 2020 assertion [22]. The protocol was approved by the International Prospective Register of Systematic Reviews (PROSPERO CRD42022298912).

### 2.2. Statistical Analysis

In order to build forest plots of continuous data and evaluate differences in PON-1 concentrations between OSA and non-OSA subjects, standardized mean differences (SMDs) and 95% confidence intervals (CIs) were used (significance level at *p* < 0.05). When necessary, means and standard deviations were extrapolated from medians and interquartile ranges [23] or from graphs using the Graph Data Extractor software.

The Q statistic (significance level at *p* < 0.10) and the I2 statistic (I2 < 25%, no heterogeneity; I2 between 25% and 50%, moderate heterogeneity; I2 between 50% and 75%, large heterogeneity; and I2 > 75%, extreme heterogeneity) were performed to measure heterogeneity of SMD values across studies [24,25]. In the case of presence of high heterogeneity, defined as I2 ≥ 50%, a random-effects model was used [25].

In order to investigate the influence of each study on the overall risk estimate, an analysis of sensitivity was performed [26].

The Begg’s adjusted rank correlation test and Egger’s regression asymmetry test (significance level at *p* < 0.05 for both) were used to evaluate the presence of publication bias, the associations between study size and magnitude of effect [27,28]. The Duval and Tweedie “trim and fill” method was also performed to test for, and eventually correct, publication bias [29]. Univariate meta-regression analyses were conducted to investigate relations between effect size and the following parameters: Age, percentage of males, body mass index (BMI), total cholesterol, LDL-cholesterol, HDL-cholesterol, triglycerides, apnoea hypopnea index (AHI), sample size and publication year. Statistical analyses were performed using Stata 14 (STATA Corp., College Station, TX, USA).

## 3. Results

### 3.1. Systematic Research and Study Characteristics

**Figure 1:**
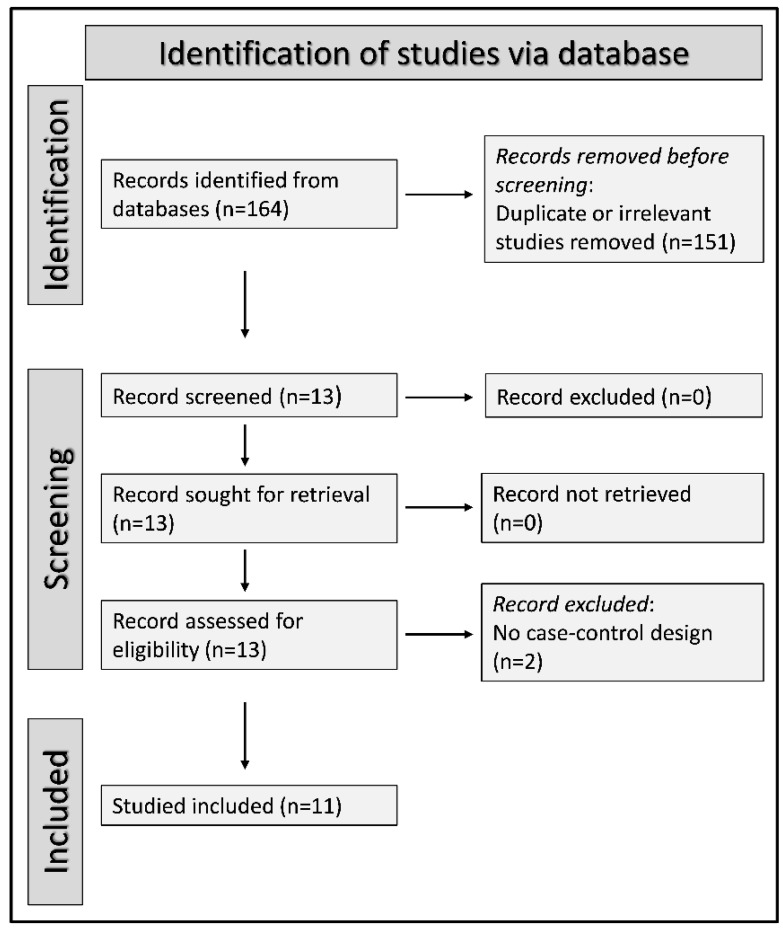
PRISMA 2020 flow diagram.

Initially, 164 potentially relevant studies were identified. After the first screening, 151 out of 164 were excluded because they were either duplicates or inappropriate. Among the remaining 13 articles, two were further excluded because they did not accomplish the inclusion criteria. Therefore, a total of 11 studies were enclosed in the meta-analysis [15,16,30,31,32,33,34,35,36,37,38]. A flow chart of the screening process is shown in Figure 1. A total of 429 OSA patients with a mean age of 49 years (84% males) and 258 non-OSA control subjects with a mean age of 47 years (74% males) were included. The characteristics of the studies, published between 2004 and 2018, are shown in Table 1.

### 3.2. Risk of Bias

In all studies included, a low risk of bias was estimated (Table 2).

### 3.3. Results of Individual Studies and Syntheses

**Figure 2:**
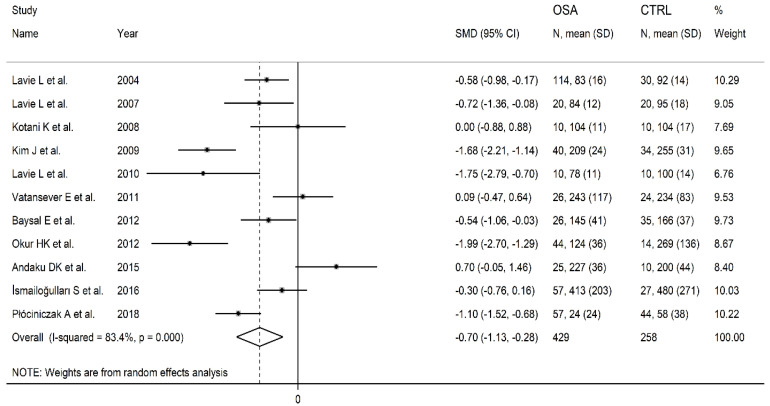
Forest plot of studies evaluating serum paraoxonase-1(PON-1) concentrations in patients with obstructive sleep apnoea(OSA) and controls.

**Figure 3:**
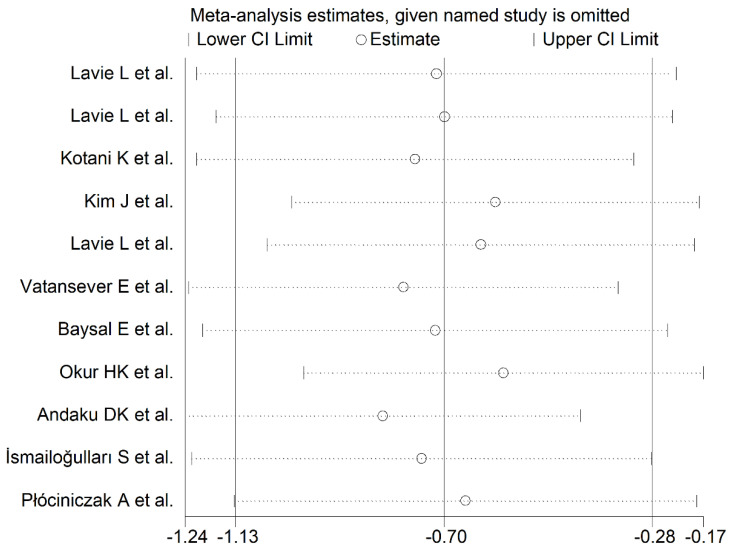
Sensitivity analysis of the association between serum PON-1 concentrations and OSA. For each study, the shown effect size (hollow circles) corresponds to an overall effect size computed from a meta-analysis excluding that study.

The forest plot reporting the PON-1 concentrations in OSA patients and control subjects is displayed in Figure 2. The results showed that in two studies [34,36], OSA patients had non-significantly higher PON-1 concentrations when compared with controls (mean difference range, 0.09 to 0.70). In one study, there were no between-group differences [31]. In the residual eight studies [15,16,30,32,33,35,37,38], OSA patients showed lower PON-1 concentrations than controls (mean difference range, −1.99 to −0.30) and the difference was statistically significant in all but one [37]. Random-effects models were used because of the extreme heterogeneity observed (I2 = 83.4%, *p* < 0.001). Pooled results indicated that PON-1 concentrations were significantly lower in OSA patients (SMD = −0.70, 95% CI −1.13 to −0.28; *p* = 0.001). Using the sensitivity analysis, the pooled SMD values were not substantially altered when individual studies were sequentially discarded (effect size range, between −0.83 and −0.58, Figure 3).

### 3.4. Publication Bias

**Figure 4:**
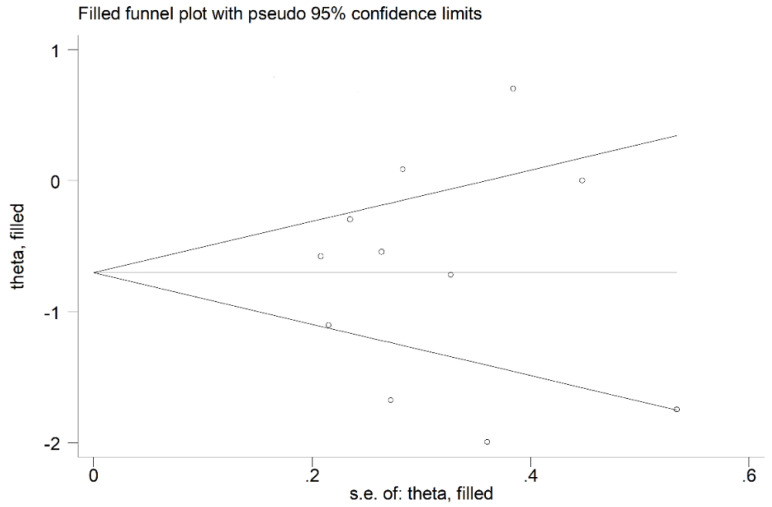
Funnel plot of studies investigating the association between serum PON-1 concentrations and OSA after trimming and filling. The enclosed circles represent the dummy studies, while free circles symbolise the genuine studies.

No publication bias was observed (Begg’s test, *p* = 1.00; Egger’s test, *p* = 0.99). Therefore, the trim-and-fill method did not detect missing studies to be included in the funnel plot (Figure 4).

### 3.5. Meta-Regression Analysis

No significant associations were detected between effect size and age (t = 0.62, *p* = 0.55), percentage of males (t = −0.124, *p* = 0.25), BMI (t = −0.55, *p* = 0.60), total cholesterol (t = 0.35, *p* = 0.74), LDL-cholesterol (t = 0.36, *p* = 0.73), HDL-cholesterol (t = 1.08, *p* = 0.32), triglycerides (t = −0.55, *p* = 0.60), AHI (t = −0.23, *p* = 0.83), sample size (t = −0.26, *p* = 0.80) and publication year (t = 0.30, *p* = 0.77).

### 3.6. Certainty of Evidence

The initial level of certainty for PON SMD values was evaluated low, due to the cross-sectional nature of the studies (rating 2, ⊕⊕⊖⊖). After considering the presence of low risk of bias in all articles (no rating change required), the high and unexplained heterogeneity (downgrade one level), the absence of indirectness (no rating change required), the relatively low imprecision (relatively narrow confidence intervals without threshold crossing, no rating change required), the moderate effect size (SMD = −0.70, no rating change required) and the absence of publication bias (no rating change required), the global level of certainty was degraded to very low (rating 1, ⊕⊖⊖⊖).

## 4. Discussion

The overproduction of ROS in OSA patients has been primarily attributed to the nocturnal intermittent hypoxia typical of the condition [4]. Several studies have reported excessive oxidative stress in untreated OSA and reduced oxidative stress burden during continuous positive airway pressure therapy [39,40]. It has been also proposed that the imbalance between pro-oxidant and antioxidant systems might contribute to the development of atherosclerotic cardiovascular disease in OSA [41]. In this context, an impaired capacity of PON-1 to preserve LDL from the oxidation of free radical has been reported in subjects with high cardiovascular risk [42]. Furthermore, PON-1 prevents endothelial dysfunction, commonly observed in OSA [43,44]. Therefore, PON-1 may be a useful marker for monitoring the oxidant–antioxidant balance in OSA patients.

The pooled SMD values observed in our study indicate a significant reduction in serum PON-1 concentrations in OSA patients when compared with non-OSA controls. Despite the extreme and unexplained between-study heterogeneity observed, the SMD values were not significantly modified in sensitivity analysis and there was no publication bias. No significant associations were observed between effect size and age, gender, BMI, total cholesterol, LDL cholesterol, HDL cholesterol, triglycerides, AHI, sample size and year of publication. Other unreported factors affecting the pro- vs. antioxidant balance, e.g., smoking history, comorbidities, use of specific medications, diet, lifestyle and genetic determinants of PON-1, may have contributed to the observed heterogeneity. In particular, the PON-1 R192Q and PON-1 L55M genetic polymorphisms have been associated with differences in PON-1 concentration and activity [45]. Despite these limitations, this meta-analysis supports the presence of a significant reduction of PON-1 concentrations in OSA.

Recently, a meta-analysis evaluating PON-1 concentrations in OSA patients was published by Fadaei et al. [46]. However, no significant differences in PON-1 concentrations were reported in a smaller number of studies, 5 vs. 11, which might explain their findings. Additionally, we conducted meta-regression analyses and assessed the certainty of evidence according to GRADE.

## 5. Conclusions

Our systematic review and meta-analysis showed significantly lower concentrations of the antioxidant PON-1 in OSA patients when compared to non-OSA controls. This supports the existence of a reduction in antioxidant protection mechanisms in OSA, likely secondary to nocturnal intermittent hypoxia. However, the significant heterogeneity observed warrants further research using rigorous standardized methods and diagnostic criteria to better establish the clinical utility of PON-1 measurements in OSA patients.

## Figures and Tables

**Table 1:** Study characteristics.

	Control				OSA				
First Author and Year, Country	N	Age Mean or Median	M/F	PON Mean ± SD (U/L)	N	Age Mean or Median	M/F	PON Mean ± SD (U/L)	AHI Events/h
Lavie L et al., 2004, Israel	30	43	27/3	92 ± 14 *	114	53	91/23	83 ± 16 *	NR
Lavie L et al., 2007, Israel	20	42	16/4	95 ± 18 *	20	42	16/4	84 ± 12 *	29
Kotani K et al., 2008, Japan	10	52	5/5	104 ± 17	10	52	5/5	104 ± 11	≥20
Kim J et al., 2009, South Korea	34	45	34/0	255 ± 31	40	45	40/0	209 ± 24	54
Lavie L et al., 2010, Israel	10	43	8/2	100 ± 14 *	10	45	8/2	78 ± 11 *	29
Vatansever E et al., 2011, Turkey	24	47	24/0	234 ± 83	26	49	26/0	243 ± 117	38
Baysal E et al., 2012, Turkey	35	47	15/20	166 ± 37	26	49	NR	145 ± 41	31
Okur HK et al., 2012, Turkey	14	49	11/3	269 ± 136	44	44	40/4	124 ± 36	37
Andaku DK et al., 2015, Brazil	10	43	10/0	200 ± 44 *	25	44	25/0	227 ± 36 *	35
İsmailoğulları S et al., 2016, Turkey	27	48	18/9	480 ± 271	57	47	45/12	413 ± 203	42
Płóciniczak A et al., 2018, Poland	44	52	23/21	58 ± 38	57	58	44/13	24 ± 24	22

Legend: AHI, apnoea-hypopnea index; NR, not reported. * U/mL.

**Table 2:** The Joanna Briggs Institute critical appraisal checklist.

Study	Were the Criteria for Inclusion in the Sample Clearly Defined?	Were the Study Subjects and the Setting Described in Detail?	Was the Exposure Measured in a Valid and Reliable Way?	Were Objective, Standard Criteria Used for Measurement of the Condition?	Were Confounding Factors Identified?	Were Strategies to Deal with Confounding Factors Stated?	Were the OUTCOMES Measured in a Valid and Reliable Way?	Was Appropriate Statistical Analysis Used?	Risk of Bias
**Lavie L et al.**	Yes	Yes	Yes	Yes	Yes	Yes	Yes	Yes	Low
**Lavie L et al.**	Yes	Yes	Yes	Yes	No	No	Yes	No	Low
**Kotani K et al.**	Yes	Yes	Yes	Yes	No	No	Yes	No	Low
**Kim J et al.**	Yes	Yes	Yes	Yes	Yes	Yes	Yes	Yes	Low
**Lavie et al.**	Yes	Yes	Yes	Yes	No	No	Yes	No	Low
**Vatansever E et al.**	Yes	Yes	Yes	Yes	Yes	Yes	Yes	Yes	Low
**Baysal E et al.**	Yes	Yes	Yes	Yes	No	No	Yes	No	Low
**Okur HK et al.**	Yes	Yes	Yes	Yes	No	No	Yes	No	Low
**Andaku DK et al.**	Yes	Yes	Yes	Yes	Yes	Yes	Yes	Yes	Low
**İsmailoğulları S et al.**	Yes	Yes	Yes	Yes	Yes	Yes	Yes	Yes	Low
**Płóciniczak A et al.**	Yes	Yes	Yes	Yes	No	No	Yes	No	Low

## Data Availability

Not applicable.
